# Ferroptosis patterns and tumor microenvironment infiltration characterization in esophageal squamous cell cancer

**DOI:** 10.3389/fgene.2022.1047382

**Published:** 2022-12-09

**Authors:** Lu-Lu Zhang, Wei-Jie Zhu, Xin-Xin Zhang, Da Feng, Xi-Cheng Wang, Ying Ding, Dong-Xia Wang, Yi-Yang Li

**Affiliations:** ^1^ Department of Molecular Diagnostics, Sun Yat-sen University Cancer Center, State Key Laboratory of Oncology in South China, Collaborative Innovation Center for Cancer Medicine, Guangzhou, Guangdong, China; ^2^ Department of Otolaryngology, Liaocheng People’s Hospital, Liaocheng, Shandong, China; ^3^ Department of Oncology, The First Affiliated Hospital of Guangdong Pharmaceutical University, Guangzhou, Guangdong, China; ^4^ Department of Radiation Oncology, Affiliated Dongguan People’s Hospital, Southern Medical University, Dongguan, Guangdong, China

**Keywords:** ferroptosis, esophageal squamous cancer, tumor microenvironment, immunotherapy, bioinformatics and biomarkers

## Abstract

**Background:** Esophageal Squamous Cell Cancer (ESCC) is an aggressive disease associated with a poor prognosis. As a newly defined form of regulated cell death, ferroptosis plays a crucial role in cancer development and treatment and might be a promising therapeutic target. However, the expression patterns of ferroptosis-related genes (FRGs) in ESCC remain to be systematically analyzed.

**Methods:** First, we retrieved the transcriptional profile of ESCC from TCGA and GEO datasets (GSE47404, GSE23400, and GSE53625) and performed unsupervised clustering to identify different ferroptosis patterns. Then, we used the ssGSEA algorithm to estimate the immune cell infiltration of these patterns and explored the differences in immune cell abundance. Common genes among patterns were finally identified as signature genes of ferroptosis patterns.

**Results:** Herein, we depicted the multi-omics landscape of FRGs through integrated bioinformatics analysis and identified three ESCC subtypes with distinct immune characteristics: clusters A-C. Cluster C was abundant in CD8^+^ T cells and other immune cell infiltration, while cluster A was immune-barren. By comparing the differently expressed genes between clusters of diverse datasets, we defined a gene signature for each cluster and successfully validated it in the TCGA-ESCC dataset.

**Conclusion:** We provided a comprehensive insight into the expression pattern of ferroptosis genes and their interaction with immune cell infiltration. Additionally, we established a gene signature to define the ferroptosis patterns, which might be used to predict the response to immunotherapy.

## Introduction

Esophageal cancer (ESCA) is an aggressive disease, ranking seventh for incidence and sixth for mortality globally in 2020 (Sung et al., 2021). Squamous cell carcinoma is the predominant subtype of ESCA in Asian countries, including China, and has a molecular profile distinct from esophageal adenocarcinoma (Napier et al., 2014; Lagergren et al., 2017; Cao et al., 2021; Sung et al., 2021). Since most patients have advanced-stage diseases at first diagnosis, even with multidisciplinary and combined treatments, including surgery, chemotherapy, and radiotherapy, the 5-year overall survival (OS) of ESCA patients remains about 20–30% (Njei et al., 2016; Lagergren et al., 2017; Zeng et al., 2018). Recent clinical trials have presented amazing therapeutic effects of immune checkpoint inhibitors, such as anti-PDL-1/PD-1 antibodies (Kato et al., 2019; Shah et al., 2019; Huang et al., 2020). However, only 20% of patients have PDL-1 expression, limiting the use of immunotherapy (Kelly, 2019). Therefore, it is imperative to develop new and effective treatments.

Ferroptosis, a newly defined form of regulated cell death, has attracted increasing attention (Shen et al., 2018; Friedmann Angeli et al., 2019; Lei et al., 2020; Li et al., 2020; Wang et al., 2020; Jiang et al., 2021). This process depends on iron and reactive oxygen species (ROS) and differs from apoptosis, necrosis, atrophy, and other types of regulated cell death in morphology, biochemistry, and genetics (Wang et al., 2020; Jiang et al., 2021). Phospholipid peroxidation is considered the hallmark of ferroptosis cascades and is regulated by the cysteine/glutathione (GSH)/glutathione peroxidase 4 (GPX4) axis, ferroptosis suppressor protein 1 (FSP1), and other GPX4-independent pathways (Li and Li, 2020; Jiang et al., 2021). The mechanisms of ferroptosis suggest its critical role in cancer development and treatment. Due to active metabolism and high ROS load, cancer cells are susceptible to oxidative turbulence, whereas oxidative stress *via* excess iron is associated with ferroptosis (Stockwell et al., 2017; Kuang et al., 2020). Multiple cancer-relevant genes and signaling pathways are involved in the process (Chu et al., 2019). A recent study has revealed that NFS1 suppression—an iron-sulfur cluster biosynthetic enzyme responsible for iron-sulfur cluster maintenance upon oxygen stress—can activate the iron-starvation response and cooperates with inhibition of glutathione biosynthesis to trigger ferroptosis in lung adenoma cells (Alvarez et al., 2017). Another study emphasized the role of ferroptosis in radiation-induced bystander effect (RIBE) (Wan et al., 2020). The activation of RIBE mainly depends on the irradiated tumor cell-derived microparticles (RT-MPs) *in vivo*, mediating the ferroptosis in tumor cells, causing immunogenic cell death, and activating macrophages. Therefore, pharmacological modulation of ferroptosis has become a promising therapeutic strategy for cancer treatment (Shen et al., 2018; Li et al., 2020).

Emerging evidence has suggested that ferroptosis may interact with the tumor microenvironment (TME) and further enhance or suppress the ability to escape immune surveillance, but the specific mechanism remains unclear (Wan et al., 2020; Wang et al., 2020; Dai et al., 2020). In pancreatic ductal adenocarcinoma, researchers have found that the extracellular release of KRAS^G12D^ during autophagy-dependent ferroptosis can drive macrophages to switch to an M2-like pro-tumor phenotype *via* STAT3-dependent fatty acid oxidation, finally promoting tumor growth (Dai et al., 2020). Additionally, the direct crosstalk between the immune system and ferroptosis has been validated. Immunotherapy-activated CD8^+^ T cells induce peroxidation in tumor cells *via* interferon-gamma, and the increased ferroptosis amplifies immunotherapy’s efficacy (Wang et al., 2019). Thus, ferroptosis might assist in promoting the antitumor effects of immunotherapy.

Nevertheless, the complete landscape of ferroptosis in ESCC remains unknown. Therefore, in the present study, we systematically evaluated the expression of ferroptosis genes and the corresponding tumor immune microenvironment characteristics in ESCC and established different ferroptosis patterns. Our current findings might be valuable for predicting the response to immunotherapy.

## Materials

### Datasets and ferroptosis-related genes (FRGs)

The gene expression data and the corresponding clinical characteristics of ESCC patients were retrieved from The Cancer Genome Atlas (TCGA) (https://portal.gdc.cancer.gov/) and Gene Expression Omnibus (GEO) (https://www.ncbi.nlm.nih.gov/geo) databases. We obtained the microarray data of GSE47404, GSE23400, and GSE53625 from GEO as the training group, which included 71, 51, and 179 samples, seperately. The detailed clinical information was presented in Table 1. The RNA sequence data of TCGA-ESCC cohort was used as the validation group with 96 samples. Raw count values were transformed into transcripts per kilobase million (TPM) values. Single nucleotide polymorphisms (SNPs) and copy number variations (CNVs) data were also downloaded from TCGA database to evaluate somatic mutations. The mutation atlas was annotated and visualized using the “maftools” R package. Tumor mutation burden was also computed for further analysis. Since all data used here is publicly available, this study did not require the approval of the local ethics committee.

**TABLE 1:** Clinical Characteristics of ESCC patients in GEO and TCGA cohort.

	GEO53625 N = 179 (%)	GEO47404 N = 71 (%)	TCGA-ESCC N = 96 (%)
Age (median)	59.6	66	61
Sex			
female	33 (18.4)	9 (12.7)	15 (15.6)
male	146 (81.6)	59 (83.1)	81 (84.4)
unkown	—	3 (4.2)	—
Location			
lower	62 (34.6)	—	39 (40.6)
middle	97 (54.2)	—	41 (42.7)
upper	20 (11.2)	—	5 (5.2)
unkown	—	—	10 (10.4)
Grade			
poorly	49 (27.4)	11 (15.5)	—
moderately	98 (54.7)	33 (46.5)	—
well	32 (17.9)	24 (33.8)	—
unkown	—	3 (4.2)	—
T stage			
T1	12 (6.7)	7 (9.9)	8 (8.5)
T2	27 (15.1)	9 (12.7)	32 (34.0)
T3	110 (61.5)	44 (62.0)	50 (53.2)
T4	30 (16.8)	8 (11.3)	4 (4.3)
unkown	—	3 (4.2)	—
N stage			
N0	83 (46.4)	—	55 (59.1)
N1	62 (34.6)	—	29 (31.2)
N2	22 (12.3)	—	6 (6.5)
N3	12 (6.7)	—	3 (3.2)
Lymph node status			
negative	—	28 (39.4)	—
positive	—	40 (56.3)	—
unknown	—	3 (4.2)	—
Stage			
I	10 (5.6)	—	—
II	77 (43.0)	—	—
III	92 (51.4)	—	—
Survival status			
dead	106 (59.2)	—	32 (33.3)
survive	73 (40.8)	—	64 (66.7)

*No clinical information was attached in GSE23400 dataset.

The FerrDb database (http://www.zhounan.org/ferrdb/current/) is a web-based consortium that provides a comprehensive and up-to-date database for ferroptosis markers, regulatory molecules, and associated diseases (Zhou et al., 2020). We identified 259 FRGs (driver: 108; suppressor: 69; marker: 111). According to the instruction of this database, the confidence level was classified into four categories based on experimental reliability and reproducibility. Among them, 120 genes (59 drivers, 1 driver/marker, 1 suppressor/marker, 1 markers, 55 suppressors, and 3 drivers/suppressors) had validated evidence with strict human tests and were finally enrolled in the current study (Supplementary Table S1).

### Protein-protein interaction (PPI) analysis

The PPIs among ferroptosis genes were identified using STRING according to the instructions. The PPI network was visualized with Cytoscape software.

### Unsupervised clustering for ferroptosis genes

Next, we performed unsupervised clustering based on the expression of ferroptosis genes to identify distinct ferroptosis patterns and classify patients for further analysis. The “ConsensuClusterPlus” R package was used to perform the clustering with 1,000 repetitions, ensuring the stability of classification. The number and stability of clusters were determined by the consensus matrix and consensus cumulative distribution function (consensus CDF).

### Differentially expressed genes (DEGs) among clusters

The “limma” and “DESeq2” R packages were applied to analyze DEGs separately in the microarray and RNA-seq datasets. The significance criterion was set as an adjusted *p*-value (FDR) < 0.05 and |log_2_ [fold change (FC)]| > 1. Differentially expressed RNAs were visualized in heatmaps and volcano plots using the “pheatmap” and “ggplot2” R packages. Considering the batch effects from different datasets, we performed differential expression gene analysis separately in each dataset, and the results were summarized in Venn plot.

### Functional and pathway enrichment analyses

To determine the biological processes (BPs), molecular functions (MFs), and cellular components (CCs) related to the ferroptosis patterns, Gene Ontology (GO) and Kyoto Encyclopedia of Genes and Genomes (KEGG) analyses were implemented based on the DEGs among subgroups using the “ClusterProfiler” R package.

Furthermore, the enrichment score of validated gene sets was estimated using the “GSVA” R package to quantify the activity of biological pathways. The ssGSEA algorithm in this package was used to estimate the relative abundance of immune cell infiltration in the TME. Based on previous studies, the gene signatures of 23 immune cells and a list of 79 immune checkpoint genes were used here to estimate immune infiltration (Supplementary Table S2) (Charoentong et al., 2017; Hu et al., 2021).

### Prediction of immunotherapy responses

Further, we assessed the immunotherapy response in the Tumor Immune Dysfunction and Exclusion (TIDE) database (http://tide.dfci.harvard.edu/) to investigate potential predictive values of ferroptosis scores. The TIDE value was supposed to be associated with the probability of immunotherapy response with a default cut-off value set to 0. However, the recommended tumor types for this database are limited to melanoma and non-small cell lung cancer (NSCLC). Hence, these results should be carefully interpreted.

### Statistical analysis

Normality was tested using the Shapiro-Wilk normality test. Next, t-tests or Wilcoxon rank-sum tests were used to compare two normally or nonnormally distributed variables, respectively. Correlation coefficients were computed *via* Spearman and distance correlation analysis. For survival analysis, we used the Kaplan-Meier method to generate the survival curves and log-rank tests to identify significant differences between groups. All statistical analyses were performed in R 4.0.3 software, and a *p* < 0.05 was considered statistically significant.

## Results

### The multi-omics landscape of ferroptosis in ESCC

**FIGURE 1:**
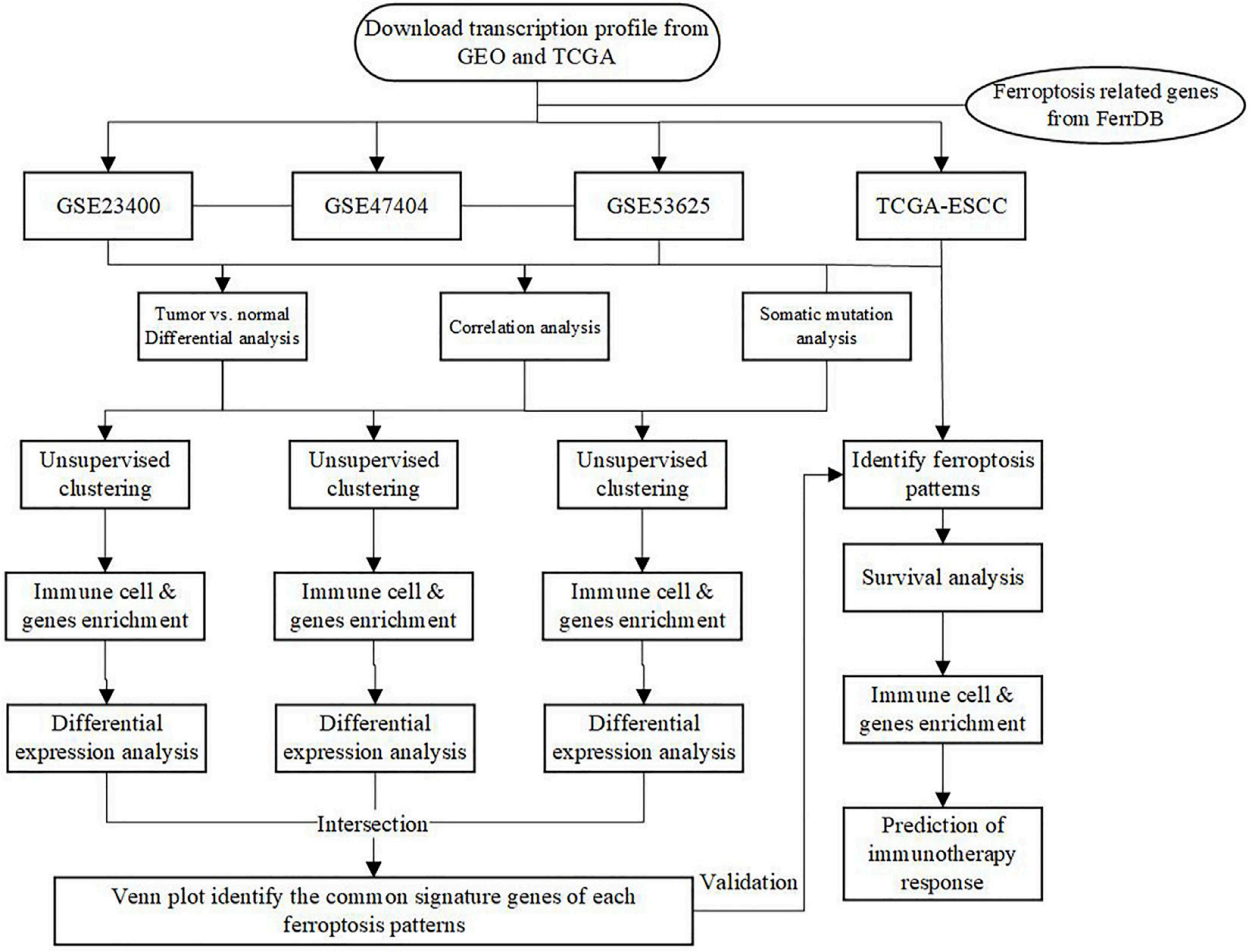
The main workflow of this study.

**FIGURE 2:**
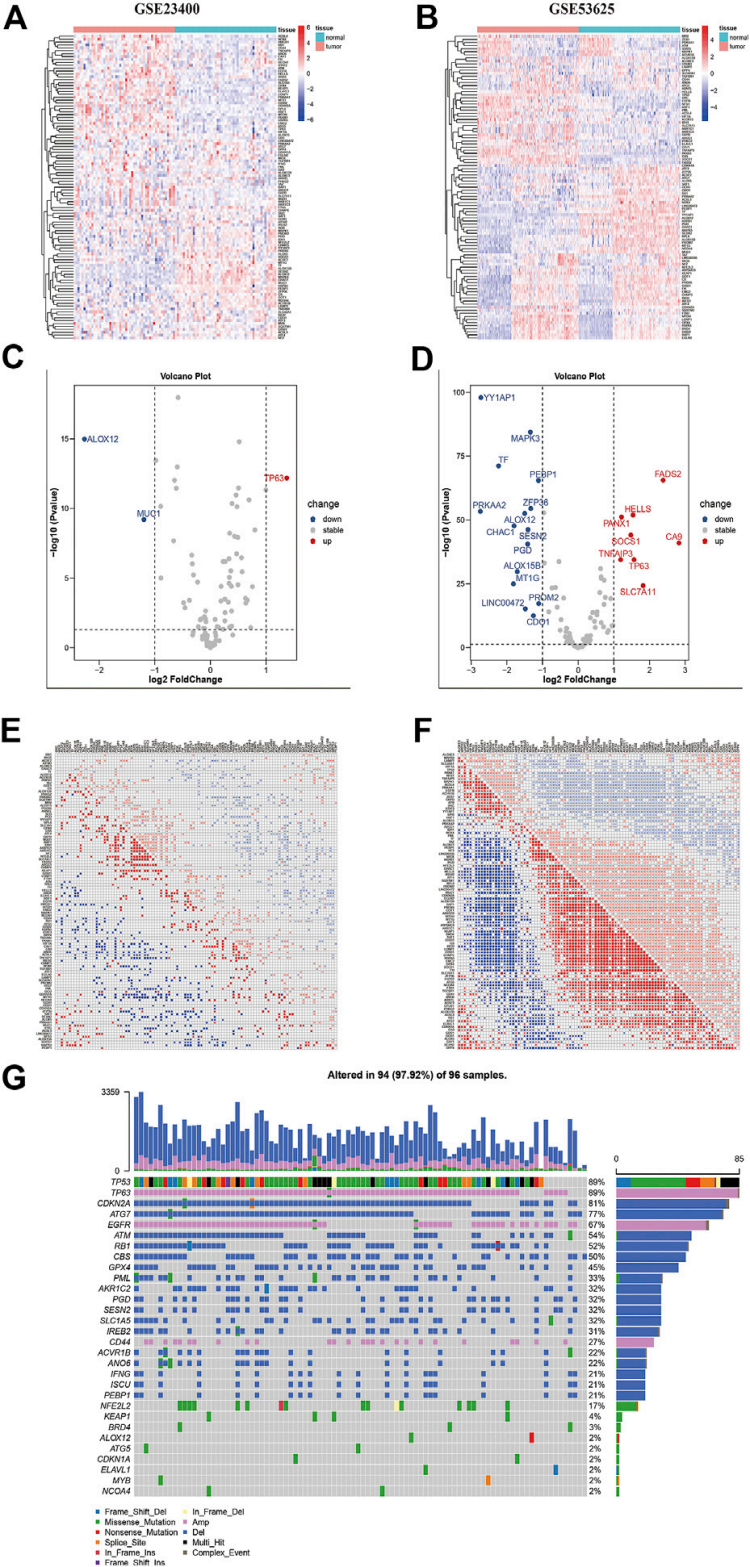
The multi-omics landscape of ferroptosis-related genes in ESCC. (A–B) Expression of 112 ferroptosis genes between normal (blue) and tumor (red) tissues in the GSE23400 **(A)** and GSE53625 **(B)** cohorts. Each column represents individual samples. The upper line represents the type of tissues. The color of each pane represents the expression level. **(C–D)** Volcano plot of differently expressed genes of GSE23400 **(C)** and GSE53625 **(D)** cohorts. Red dots represent upregulated genes, blue dots represent downregulated genes, and black dots represent genes that do not differ. **(E–F)** Correlation heatmap between ferroptosis genes in GSE23400 **(E)** and GSE53625 **(F)** cohorts. Red dots represent positive correlations, blue dots represent negative correlations, and blank represents no significant correlations. Numbers in the pane represent coefficients. **(G)** Mutation frequency of ferroptosis genes in the TCGA-ESCC cohort. Each column represents an individual patient. The number on the right indicates the mutation frequency in each regulator gene. The right barplot showed the proportion of each variant type.

The main workflow of our research was presented in Figure 1. Herein, we depicted the multi-omics landscape of FRGs genes using integrated bioinformatics analysis. First, we explored the expression of FRGs between ESCC and adjacent normal tissues in the GSE53625 and GSE23400 cohorts. Distinct ferroptosis gene expression was detected between ESCC and adjacent normal tissues (Figures 2A,B). We identified 24 differently expressed ferroptosis genes in the GSE53625 and GSE23400 cohorts with |log_2_FC| > 1 and FDR >0.05 (Figures 2C,D and Supplementary Table S3). Among these genes, 11 driver genes (PGD, TF, ALOX12, ALOX15B, MAPK3, PEPB1, CDO1, CHAC1, LINC00472, PRKAA2, and YY1AP1) were downregulated in tumor tissues, and five suppressor genes (SLC7A11, HELLS, TP63, FADS2, and CA9) were upregulated. These results indicated the ferroptosis resistance nature of ESCC samples. Next, we also conducted a correlation analysis among these ferroptosis genes (Figures 2E,F, Supplementary Figure S1). In the GSE53625, GSE47404, and GSE23400 cohorts, we detected a close relationship among G6PD, PGD, SLC7A11, ABCC1, and AKR1C3. The PPI network showed that TP53, HIF1A, STAT3, and EGFR had widespread interactions with the other genes (Supplementary Figure S2).

Further, we summarized the incidence of CNVs and somatic mutations of 116 ferroptosis regulators in the TCGA-ESCC cohort (Figure 1G). TP63, EGFR, and CD44 displayed prevalent CNV amplification, while CDKN2A, ATG7, ATM, RB1, CBS, GPX4, PML, and PGD had widespread depletion. CNVs occurred in most samples (97.9%), ranking the most common genetic alteration. Regarding single nucleotide variants, TP53 exhibited the highest mutation frequency, followed by NFE2L2, KEAP1, BRD4, and PML, with missense mutations representing the most common mutation type.

**FIGURE 3:**
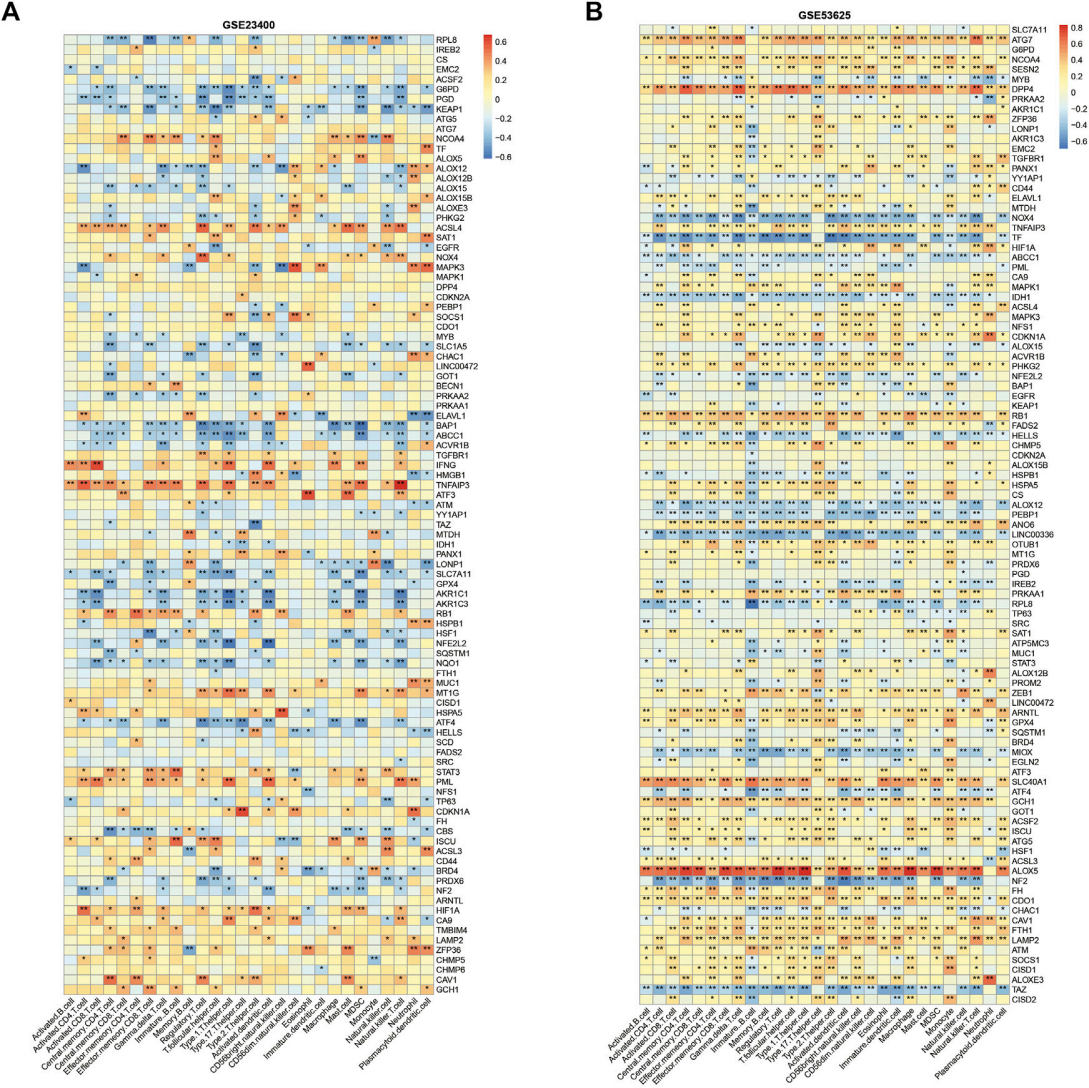
Immune correlation of ferroptosis genes in ESCC. Correlation heatmap between ferroptosis genes and immune cells in GSE23400 **(A)** and GSE53625 **(B)** cohorts. Red indicates positive correlations, and blue indicates negative correlations.

We also assessed the immune cell infiltration in bulk tumor samples using the ssGSEA algorithm to explore the roles of ferroptosis genes in immune regulation. In the GSE53625 cohort, ALOX5, DPP4, ATG7, and SLC40A1 had a strong positive correlation with most immune cell infiltration, including CD4^+^ T, CD8^+^ T, and nature killer cells, while other genes (TF, NOX4, NF2, TAZ, ALOX12, and LINC00336) presented opposite relationship with immune cell infiltration (Figure 3A). In GSE23400, TNFAIP3 and IFNG were correlated with activated CD8^+^ T cell infiltration, and NF2, ALOX12, MAPK3, AKR1C1, and AKR1C3 were associated with the suppression of activated CD8^+^ T cells (Figure 3B).

Altogether, these results depicted the multi-omics landscape of FRGs, with significant genetic alteration and expression heterogeneity between normal and tumor samples.

### Identification of different ferroptosis patterns

**FIGURE 4:**
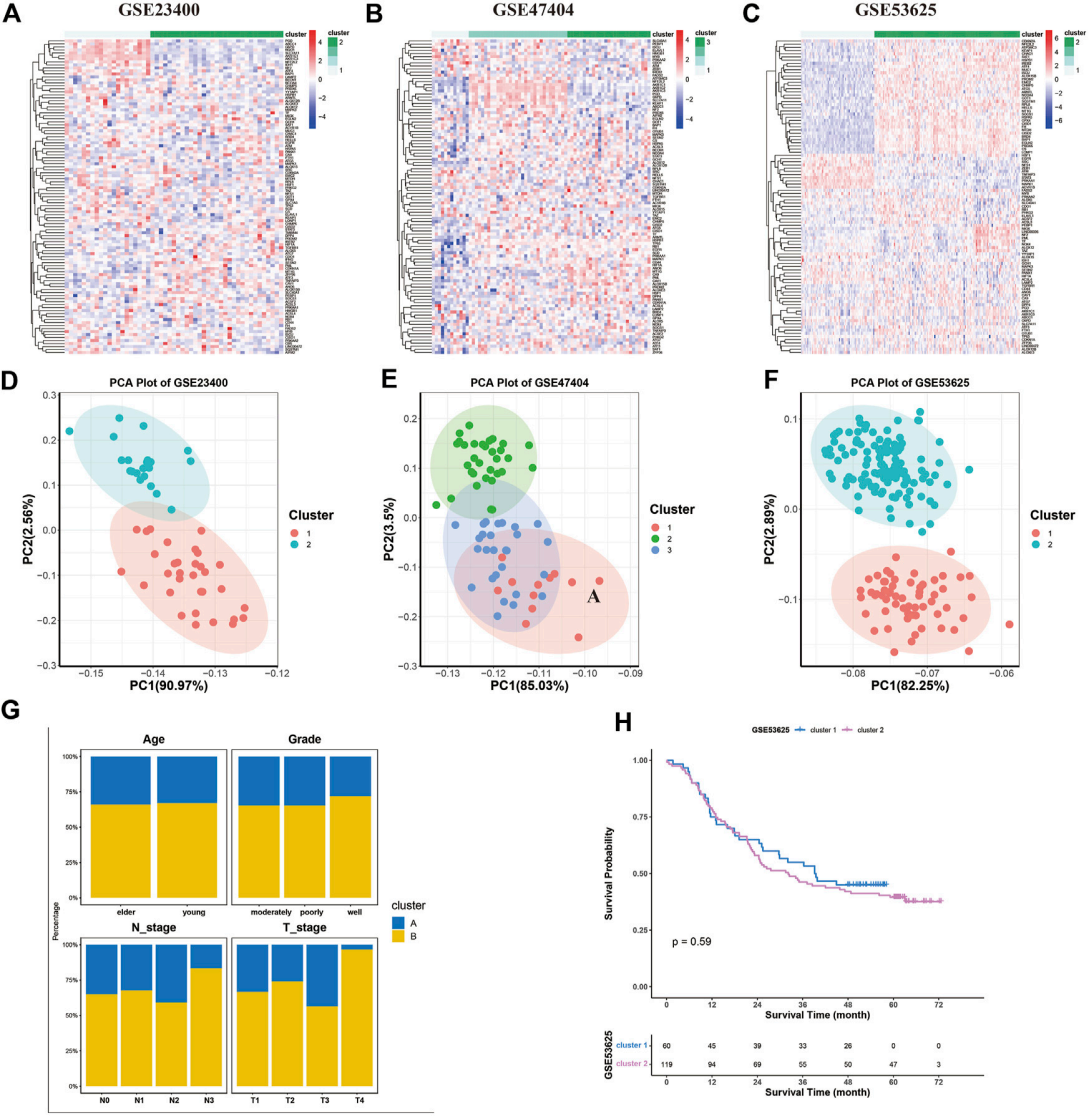
Ferroptosis patterns in the ESCC cohort. **(A–C)** Unsupervised clustering of ferroptosis genes in GSE23400 **(A)**, GSE47404 **(B)**, and GSE53625 **(C)**. The color of each pane represents the expression level with red indicating high expression, and blue indicating low expression. **(D–F)** Principal component analysis (PCA) for the transcriptome profiles of three ferroptosis patterns in GSE23400 **(D)**, GSE47404 **(E)**, and GSE53625 **(F)**. There is a remarkable difference in transcriptome between different ferroptosis patterns. **(G)** Stacked bar plot of age, grade, T stage, and N stage between clusters of the GSE53625 cohort. The ferroptosis clusters had similar age, grade, and N stage distribution, except of T stage. **(H)** Kaplan–Meier curves of two ferroptosis clusters in the GSE53625 cohort (*p* = 0.59).

Further, we used unsupervised clustering to explore ferroptosis patterns based on the expression of ferroptosis genes. Two or three clusters were determined in GSE53625, GSE23400, and GSE47404 datasets (Figures 4A–C). The CDF curve plot and principal component analysis (PCA) verified the rationality of the grouping (Figures 4D–F, Supplementary Figure S3). The heatmaps showed that, compared to cluster 1 of GSE53625, the expression of most ferroptosis genes was elevated in cluster 2, similar to clusters 1 and 2 of GSE47404. In GSE23400, cluster 1 differed from cluster 2 in the expression of AKR1C1, AKR1C2, AKR1C3, G6PD, PGD, SLC7A11, ABCC1, PML, CAV1, and MT1G. A similar phenomenon was also observed between clusters 2 and 3 of GSE47404. Thus, we hypothesized that three different ferroptosis patterns existed: ferroptosis clusters A, B and, C, corresponding to GSE47404 cluster 1 (or GSE53625 cluster 1), GSE47404 cluster 2 (or GSE53625 cluster 2/GSE23400 cluster 1), GSE47404 cluster 3 (or GSE23400 cluster 2).

Next, we investigated the relationship between clusters and matching clinical information, including age, grade status, clinical stage, and survival. Compared to cluster A, patients in cluster B tended to be in a more advanced stage. The two ferroptosis clusters did not differ in age, grade, and N stage distribution (Figure 4G). The survival analysis showed similar survival between clusters A and B (Figure 4H).

### Correlation between ferroptosis patterns and immune infiltration

**FIGURE 5:**
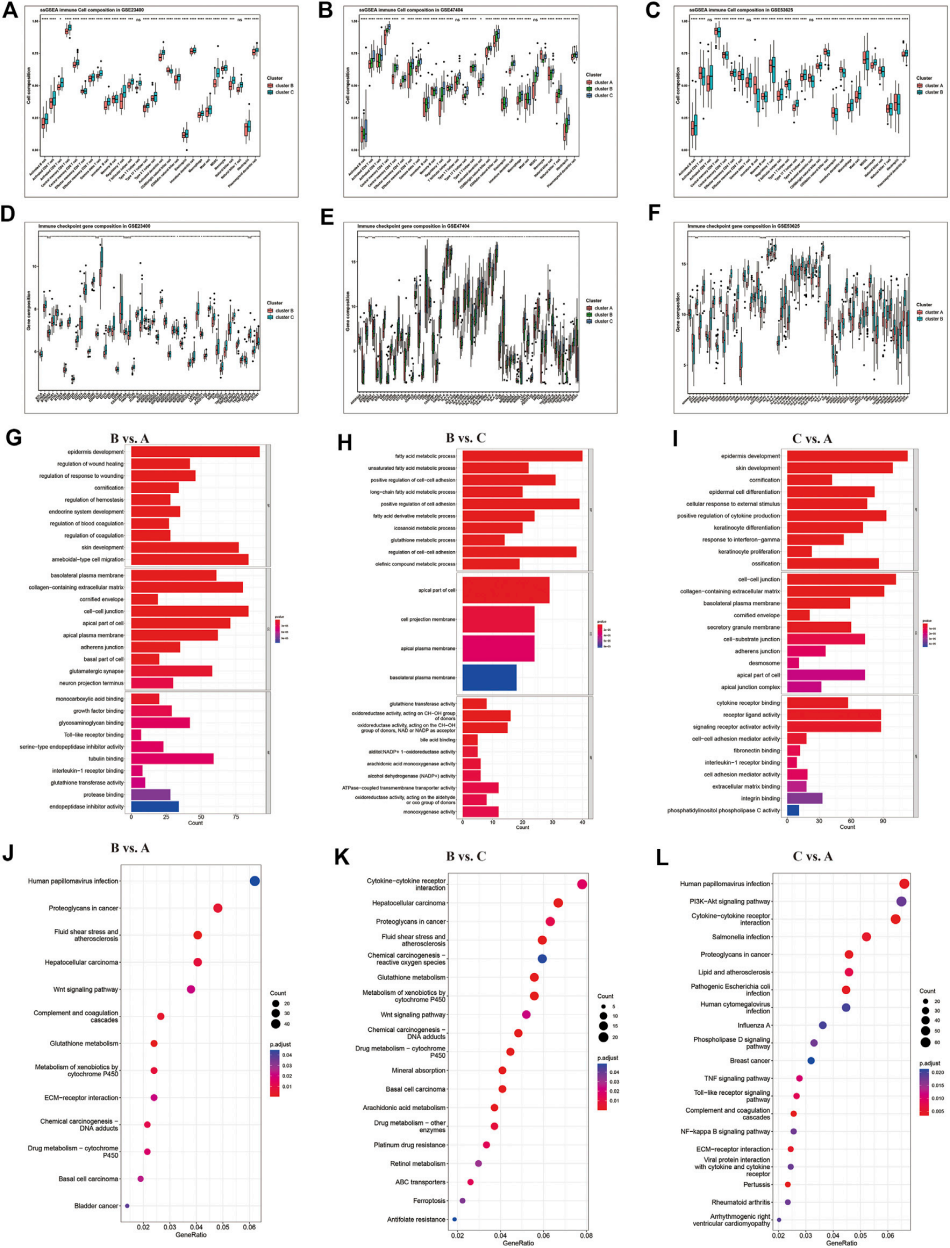
Differential immune characteristics among ferroptosis patterns. **(A–C)** Relative enrichment of 23 immune cells and **(D–F)** immune checkpoint genes in ferroptosis clusters of GSE23400, GSE47404, and GSE53625. The upper and lower ends of the boxes represent the interquartile range of values. The lines in the boxes represent the median value, and the black dots represent outliers. The asterisks represent the *p*-values (* <0.05; ** <0.01; *** <0.001; ns, no significance). **(G–I)** Gene Ontology (GO) and Kyoto Encyclopedia of Genes and **(J–L)** Genomes (KEGG) pathways analyses depicted the enriched pathways of ferroptosis-related genes: cluster B vs. cluster A, cluster B vs. cluster C, and cluster C vs. cluster A.

We also found that the clusters exhibited significant heterogeneity in immune cell infiltration and immune checkpoints gene enrichment (Figure 4). Compared to cluster A and B, cluster C displayed the most immune cell infiltration (Figures 5A–C), and a higher expression of immune checkpoint genes (Figures 5D–F), including PD-1, PD-L1, and CTLA-4. Cluster C was abundant in most immune cells, including activated CD4^+^ T cells, activated CD8^+^ T cells, activated dendritic cells, macrophages, and natural killer cells. On the other hand, cluster A presented reduced immune cell infiltration. All three GEO datasets presented similar results. Hence, cluster C was characterized by immune abundance, whereas cluster A by immune barren. Considering the close relationship with immune infiltration, we proposed that ferroptosis patterns can potentially predict immunotherapy’s anticancer efficacy.

The KEGG and GO enrichment analyses indicated that several immune-related KEGG pathways and GO annotations were enriched among clusters, such as cytokine-cytokine receptor interaction and PI3K-Akt signaling pathway involved in immune cell activation (Figures 5G–L). Altogether, these results demonstrated that ferroptosis might play an important role in immune regulation and cell proliferation in the TME.

### Molecular characteristics of ferroptosis patterns

To explore the characteristic genes of each cluster, we finally detected 3,742 DEGs in the GSE47404, 6,797 DEGs in the GSE53625, and 83 DEGs in the GSE23400 datasets. Among them, the AKR1C3 gene was the common ferroptosis DEG, presenting a vital role in clustering (Supplementary Figure S4).

**FIGURE 6:**
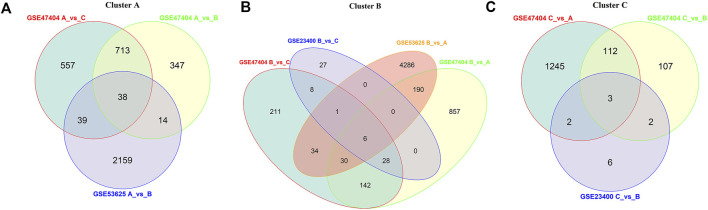
Gene signatures for each cluster. Based on the results from different datasets, venn diagrams showed the common upregulated DEGs of clusters **(A)**, **(B)**, and **(C)**.

In GSE47404, ferroptosis cluster A was characterized by upregulation of 751 DEGs compared to clusters B and C, while in the GSE53625 cohort, cluster A displayed a remarkable increase in 2,250 DEGs. We intersected the characteristic genes of cluster A and finally found 38 genes, including PRTG, KIT, PROX1, and DMD (Figure 6A).

Compared to clusters A and C, ferroptosis cluster B of the GSE47404 cohort presented elevated expression of 206 DEGs. However, in GSE23400 and GSE53625 datasets, only six DEGs were upregulated in cluster B, including AKR1C3, ALDH3A1, TMEM116, PIR, TKT, and GCLC. Among them, AKR1C3 was the only FRG and was considered the characteristic gene of cluster B (Figure 6B).

As described above, we investigated the common DEGs elevated in cluster C. In the three datasets, cluster C displayed higher BST2, EREG, and MMP13 expressions than the other two clusters. Therefore, BST2, EREG, and MMP13 were identified as the characteristic genes of cluster C (Figure 6C).

Finally, we compared the clusters of various datasets and defined a gene signature to distinguish ferroptosis patterns. We inferred that the different expression levels of signature genes might represent different ferroptosis patterns.

### Validation of the key ferroptosis genes in ferroptosis clustering

**FIGURE 7:**
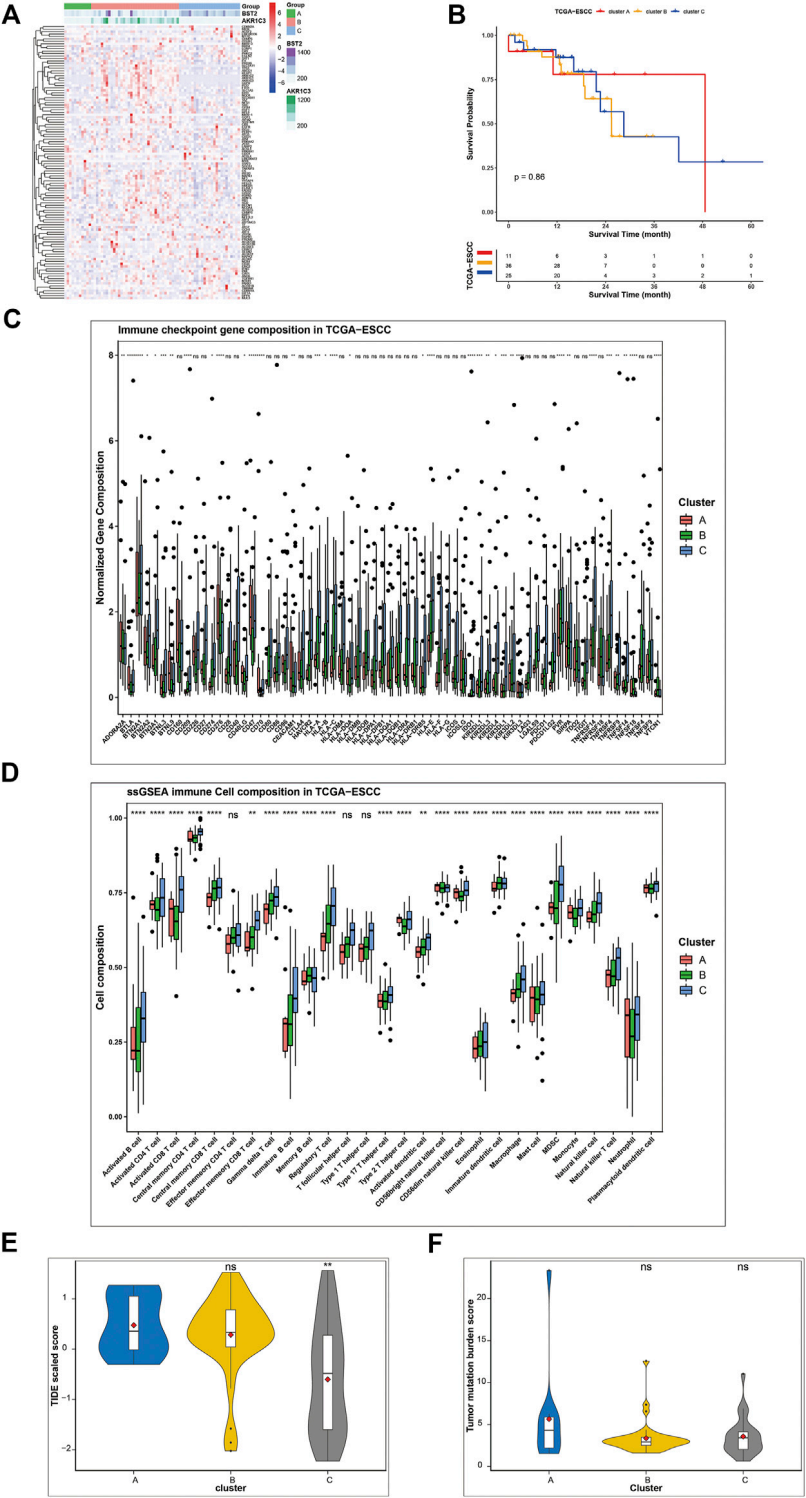
Validation of gene signatures in TCGA-ESCC cohort. **(A)** Three patterns were well identified based on the expression of signature genes. Cluster B was characterized by high expression of most ferroptosis genes, especially AKR1C3, while cluster C featured BST2 and certain genes. **(B)** Survival analysis of different ferroptosis clusters (*p* = 0.87). **(C)** Expression of checkpoint genes, **(D)** relative abundance of immune cells, **(E)** TIDE value, and **(F)** TMB score were compared among the three clusters. The asterisks represent the *p*-values (* <0.05; ** <0.01; *** <0.001; ns, no significance) (*p < 0.05, **p < 0.01,***p < 0.001, ****p < 0.0001).

To validate our results, we recruited TCGA-ESCC cohort as the validation group. We set the median expression of signature genes as the cutoff values. Based on AKR1C3 and BST2 expression, all samples of TCGA-ESCC were classified into three subgroups (Figure 7A): AKR1C3 high expression (corresponding to cluster B), AKR1C3 low and BST2 high expression (corresponding to cluster C) and both genes low expression (corresponding to cluster A) groups. Although no clinical stage or survival difference was observed, the clusters presented a significantly different immune landscape (Figure 7B). Cluster C was closely related to the infiltration level of most immune cells, such as activated CD4^+^ T cells, activated CD8^+^ T cells, immature B cells, and central memory CD8^+^ T cells, and abundant in immune gene expressions, including the common immune checkpoints PDL-1, CTLA4, and PD-1, which might be more beneficial for immunotherapy in contrast to clusters B and C (Figures 7C,D). Furthermore, we use TIDE value to predict the immune response and found that cluster C had relative lower TIDE score, which meant better response to immunotherapy (Figure 7E). As for tumor mutation burden, the three clusters shared similar TMB score (Figure 7F).

According to previous literatures, gene AKR1C3 and BST2 play an important role in malignancies and drug resistance with involvement of a range of signal pathways, including the PI3K/Akt, MAPK, ERK, and NF-κB signaling pathways (Kuang et al., 2017; Mahauad-Fernandez and Okeoma, 2017; Liu et al., 2020; Xu et al., 2020). In current research, we performed bioinformatic analysis to explore the potential function of BST2 and AKR1C3 gene (Supplementary Figure S5). GSEA revealed that AKR1C3 gene was linked with carcinogenesis, while BST2 was associated with signal transduction and phagocytosis. Survival analysis indicated no significant survival difference between high and low expression of BST2 and AKR1C3 gene.

## Discussion

The number of published studies on ferroptosis has increased in recent years. Additionally, various studies have addressed the vital role of ferroptosis in cancer development and treatment, and many cancer-relevant genes and signaling pathways have been identified (Stockwell et al., 2017; Chu et al., 2019; Dai et al., 2020; Kuang et al., 2020; Lei et al., 2020). However, most previous studies focused on a single ferroptosis regulator or a prognostic ferroptosis gene signature, and the comprehensive landscape of integrated FRGs has not yet been investigated (Chen et al., 2021; Lu et al., 2021; Song et al., 2021). In current research, we systymatically described the multi-omics landscape of FRGs genes, unveiled the distinct ferroptosis gene expression pattern, as well as its interaction with immune microenvironment, and finally identified characteristic genes of each ferroptosis patterns.

Herein, we screened out 116 validated FRGs from the FerrDB and investigated their transcriptomic and genomic profile in ESCC samples from the GEO and TCGA databases. The expression pattern heterogeneity between tumor samples and normal tissues indicated the resistance of ESCC tumors to ferroptosis. Additionally, genetic variations of FRGs were common in ESCC samples, which may initiate or suppress ferroptosis. Among these genetic variations, TP53 mutation was the most frequent event and has been proved to be involved in ferroptosis *via* SLC7A11 inhibition, independent of the traditional GTX4 pathway. According to the detailed serial analysis from Gu et al., TP53 can potentiate ferroptosis by suppressing the transcription of the Xc-system subunit SLC7A11 and contribute to the tumor suppressive function *in vitro* and *in vivo* (Chu et al., 2019). After TP53, NFE2L2 exhibited the most frequent gene mutation. NFE2L2 is a nuclear transcription factor vital in counteracting oxidative and electrophilic stresses through transcribing antioxidant genes (Ryoo and Kwak, 2018; Kuang et al., 2020). Besides, NFE2L2 contributes to lipid metabolism, iron homeostasis, and other pathways, which interact with the ferroptosis cascade (Kuang et al., 2020; Song et al., 2021). CDKN2A was another common gene with CNV. Deleting CDKN2A can act as an oxidative stress-induced genetic alteration, inhibit cyclin-dependent kinases from promoting DNA replication, and is involved in activating the TP53 signaling pathway (Zhao et al., 2016; Serra and Chetty, 2018). Moreover, CDKN2A is also recognized as a cuproptosis gene.

We identified three distinct ferroptosis clusters characterized by different immune environments based on the transcriptional pattern of ferroptosis genes. Ferroptosis cluster C was characterized by high infiltration of almost all kinds of immune cells and enriched in immune checkpoint genes. In contrast, cluster A presented decreased immune cell infiltration and a lack of immune checkpoint genes. The functional analysis validated that the immune phenotypes of cluster C were linked with several immune activation pathways. This heterogeneity might predict different responses to immunotherapy. PD-1/PDL-1 and CTLA4 inhibitors have been approved for clinical treatment, but only patients with high expression of PD-1, PDL-1, or CTLA4 could benefit from immunotherapy. According to the current study, ferroptosis clusters distinguished the gene expression into three levels and were associated with different responses to PD-1/PDL-1 blockade. Thus, we inferred that ferroptosis patterns are potential biomarkers for immunotherapy.

Furthermore, we explored the ferroptosis-related DEGs among clusters and identified a set of characteristic genes for each cluster. For example, cluster B demonstrated significant upregulation of AKR1C3 compared to the other two clusters, while cluster C was characterized by elevated expression of BST2, EREG, and MMP13. Different from clusters B and C, cluster A was characterized by a 38 gene set including PRTG, KIT, PROX1, and DMD. We successfully defined the clusters with these characteristic genes. A clustering algorithm was developed based on the characteristic gene expression. Using the median expression as the cut-off, we classified ESCC samples of TCGA dataset into ferroptosis cluster A with low expression of AKR1C3 and BST2, ferroptosis cluster B with high expression of AKR1C3, and ferroptosis cluster C with low expression of AKR1C3 and high expression of BST2. The three patterns displayed distinct immune phenotypes, similar to GEO exploration cohorts. Cluster C might have better response to immunotherapy. Compared to scores derived from PCA or GSVA algorithms, our current clustering algorithm showed an advantage in omitting complex computation and relying less on gene distribution of individual cohorts, which facilitates clinical application.

However, our current study also has some limitations. The main shortcoming of this study was the limited number of clinical samples used for validation, which requires further investigation. Moreover, cell experiments are needed to validate our hypotheses. Based on findings derived from public data, we will subsequently explore the mechanisms of vital FRGs in immune activation.

In summary, we provided a comprehensive insight into the expression pattern of ferroptosis genes and their interaction with TME immune cell infiltration. We demonstrated that different ferroptosis patterns could distinguish the landscape of the TME immune cell infiltration and immune checkpoint genes. Finally, we established a clustering algorithm to define ferroptosis patterns. These integrated analyses highlighted the vital role of ferroptosis in immune activation in ESCC, which might also contribute to guiding immunotherapy strategies.

## Data Availability

The datasets presented in this study can be found in online repositories. The names of the repository/repositories and accession number(s) can be found in the article/Supplementary Material.
