# Supplementary figures and images for: Ferroptosis patterns and tumor microenvironment infiltration characterization in esophageal squamous cell cancer
Source: Front Genet. 2022 Dec 9;13:1047382. doi: 10.3389/fgene.2022.1047382 (PMC9780266; doi:10.3389/fgene.2022.1047382)

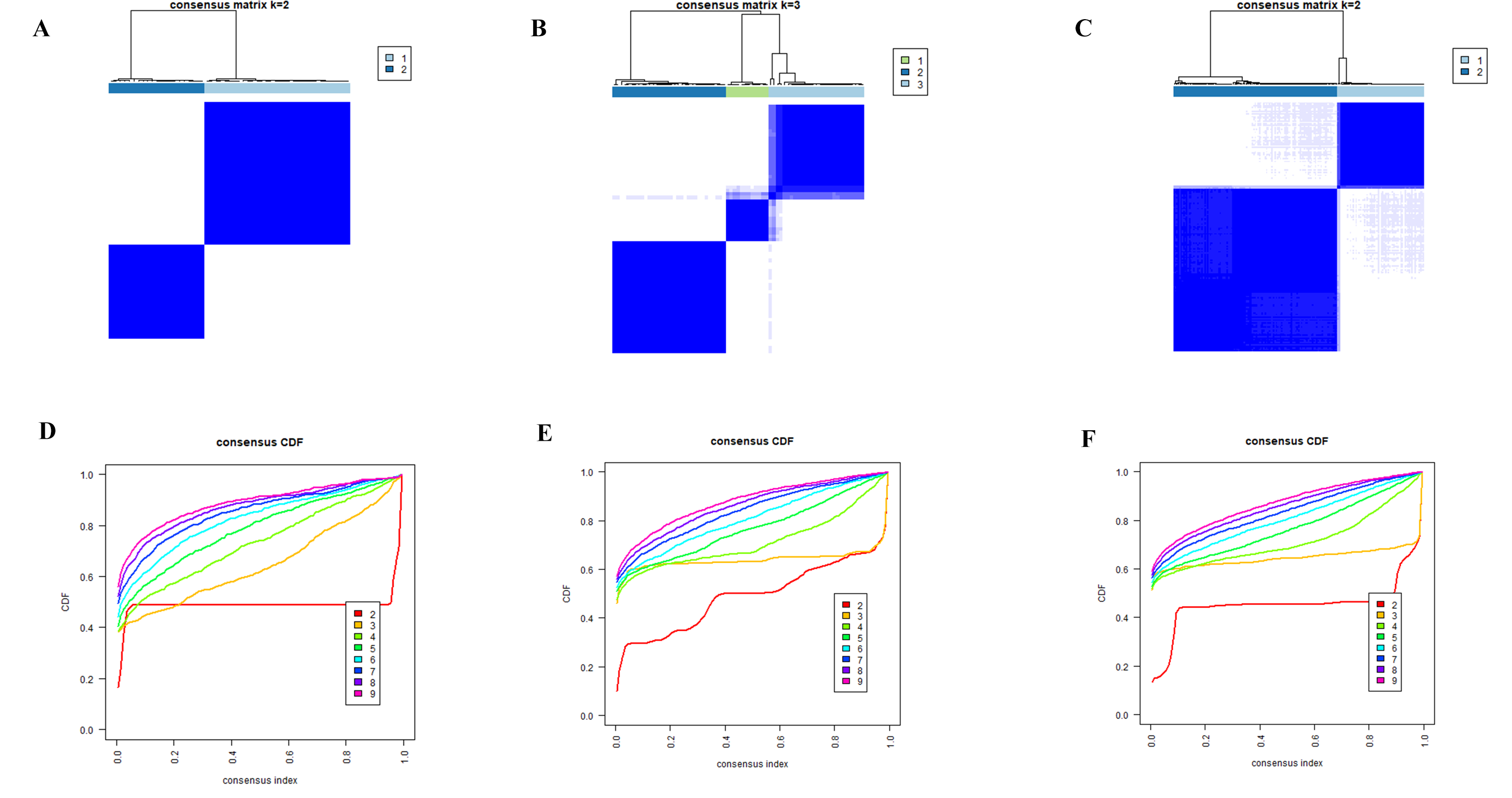

Supplement: Supplementary file 2 [file Image3.TIF]

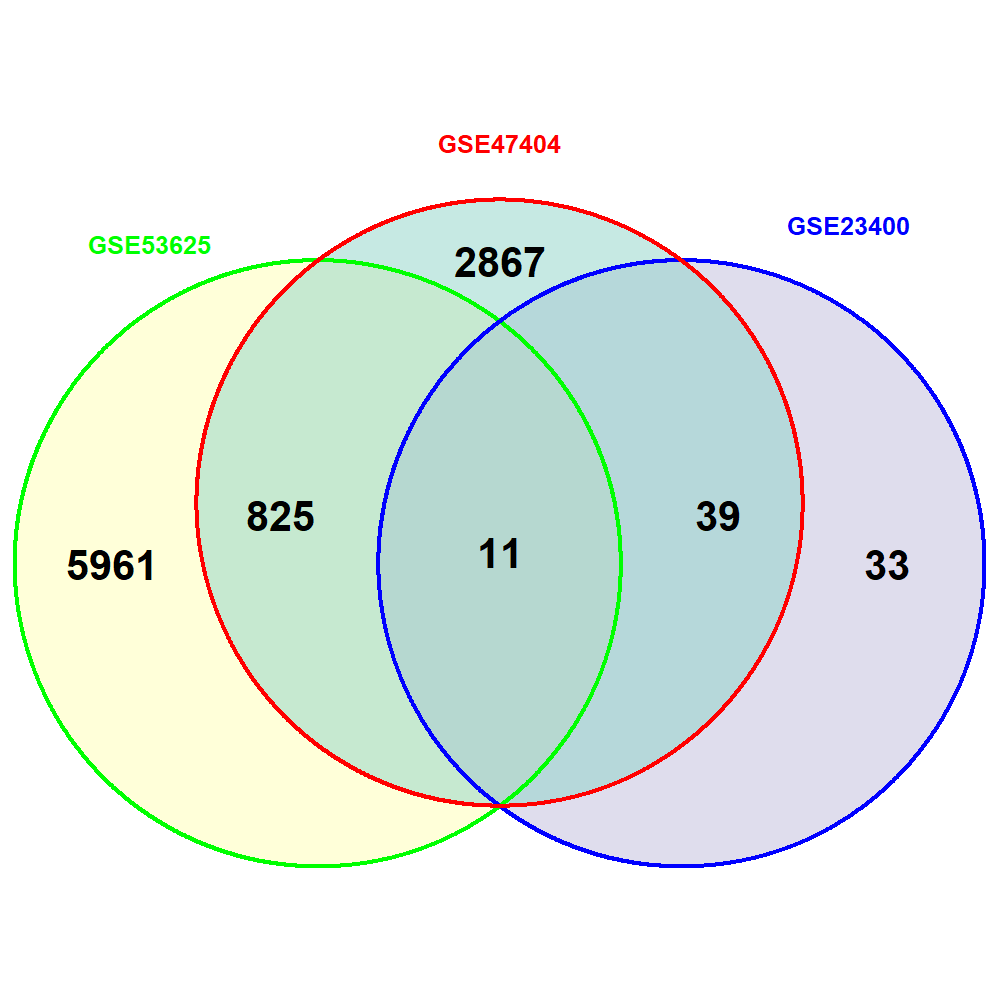

Supplement: Supplementary file 3 [file Image4.TIF]

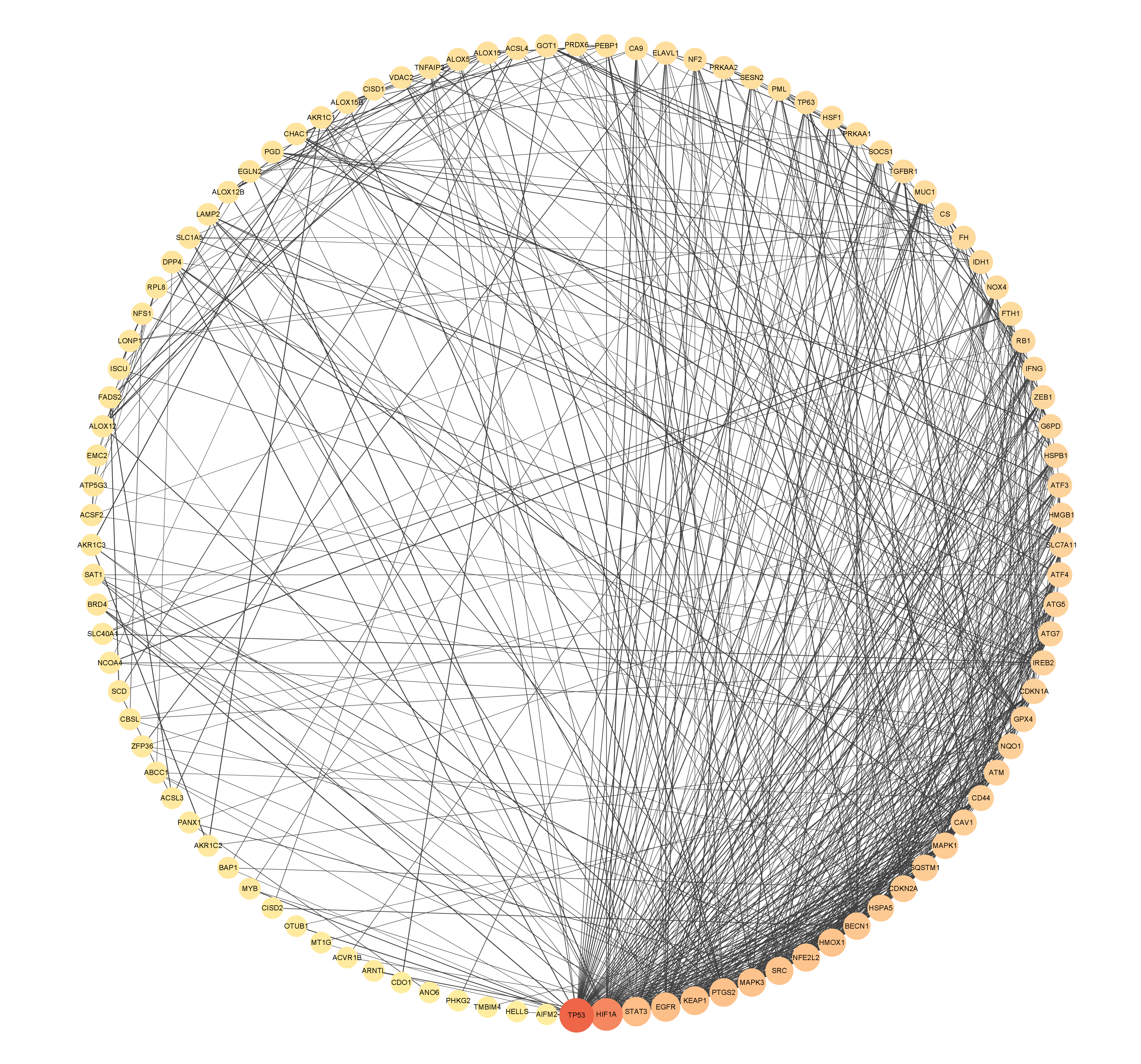

Supplement: Supplementary file 4 [file Image2.TIF]

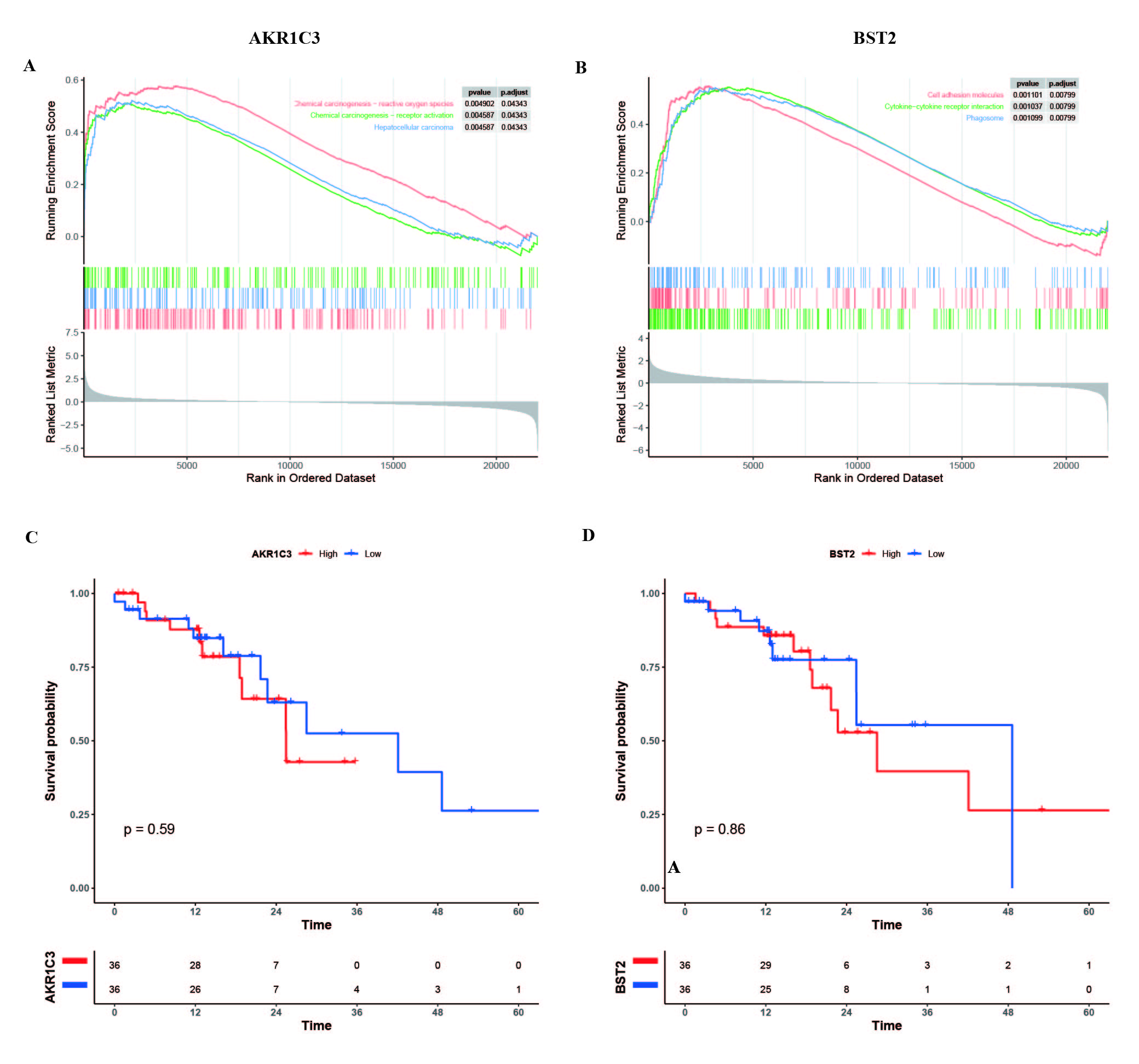

Supplement: Supplementary file 5 [file Image5.JPEG]
